# HbF Levels in Sickle Cell Disease Are Associated with Proportion of Circulating Hematopoietic Stem and Progenitor Cells and CC-Chemokines

**DOI:** 10.3390/cells9102199

**Published:** 2020-09-29

**Authors:** Caterina P. Minniti, Seda S. Tolu, Kai Wang, Zi Yan, Karl Robert, Shouping Zhang, Andrew S. Crouch, Joan Uehlinger, Deepa Manwani, Eric E. Bouhassira

**Affiliations:** 1 Department of Medicine, Hematology, Albert Einstein College of Medicine, Bronx, NY 10461, USA; caterina.minniti@einsteinmed.org (C.P.M.); stolu@montefiore.org (S.S.T.); Andrew.crouch@einstein.yu.edu (A.S.C.); 2 Department of Cell Biology, Albert Einstein College of Medicine, Bronx, NY 10461, USA; kai.wang@tarabiosystems.com (K.W.); zi.yan@einsteinmed.org (Z.Y.); karl.roberts@einsteinmed.org (K.R.); Shouping.Zhang@einsteinmed.org (S.Z.); 3 Department of Pathology, Division of Transfusion Medicine, Montefiore Health System, Bronx, NY 10467, USA; juehling@montefiore.org; 4 Pediatric Hematology/Oncology/Marrow and Blood Cell Transplantation, Montefiore Health System, Bronx, NY 10467, USA; dmanwani@montefiore.org

**Keywords:** sickle cell disease, hematopoietic stem and progenitor, chemokine, fetal hemoglobin

## Abstract

The concentration of circulating hematopoietic stem and progenitor cells has not been studied longitudinally. Here, we report that the proportions of Lin-CD34+38- hematopoietic multipotent cells (HMCs) and of Lin-CD34+CD38+ hematopoietic progenitors cells (HPCs) are highly variable between individuals but stable over long periods of time, in both healthy individuals and sickle cell disease (SCD) patients. This suggests that these proportions are regulated by genetic polymorphisms or by epigenetic mechanisms. We also report that in SCD patients treated with hydroxyurea, the proportions of circulating HMCs and HPCs show a strong positive and negative correlation with fetal hemoglobin (HbF) levels, respectively. Titration of 65 cytokines revealed that the plasma concentration of chemokines CCL2, CCL11, CCL17, CCL24, CCL27, and PDGF-BB were highly correlated with the proportion of HMCs and HPCs and that a subset of these cytokines were also correlated with HbF levels. A linear model based on four of these chemokines could explain 80% of the variability in the proportion of circulating HMCs between individuals. The proportion of circulating HMCs and HPCs and the concentration of these chemokines might therefore become useful biomarkers for HbF response to HU in SCD patients. Such markers might become increasingly clinically relevant, as alternative treatment modalities for SCD are becoming available.

## 1. Introduction

Sickle cell disease (SCD) is a single-point mutation from glutamic acid to valine that results in pleomorphic clinical complications caused by polymerization of hemoglobin S (HbS) and its downstream vaso-occlusive complications, many of which increase with age [1,2]. Chronic hemolysis and the resultant anemia, punctuated by recurrent episodes of pain, result in early end-organ damage, poor quality of life, and decreased life expectancy [3]. SCD currently affects 25 million people worldwide, a number projected to increase by 30% in 2050 [4,5].

The current therapies for SCD involve treatment with either hydroxyurea (HU) or transfusion. Transplantation and gene therapy are exciting curative strategies, but their large-scale applications are unlikely to occur in the short term, for the vast majority of patients worldwide, because of their complexity, difficulties in finding matched donors, high risks, and costs. Pharmacological interventions are therefore likely to continue to play an important therapeutic role in the foreseeable future. In addition to HU, several drugs based on decreasing HbS polymerization are in development or were recently approved [6,7]. Whether they will prove complementary or more efficacious than the current standard of therapy with HU is uncertain at this time. Understanding the mechanism of action of HU and finding additional drugs to increase fetal hemoglobin production, therefore, remain priorities in the treatment of SCD.

HU was the first drug approved by the food and drug administration (FDA), for clinical use in patients with SCD, to induce HbF. However, clinical response remains variable [8,9] and many patients do not reach a clinically significant increase in HbF once the maximum tolerated dose is determined [10,11]. Recently, Bcl11a was shown to be a critical regulator of HbF expression in adults, and a SNP within the Bcl11a gene was found to be correlated with the HU response [8,12,13]. Genetic polymorphisms at the α- and β-globin loci, particularly in the γ-globin promoters, are also known modifiers of HbF expression [14,15]. However, no combination of predictors can completely explain the variability in HbF induction levels after HU treatment, suggesting additional contributing factors.

At the molecular level, HU was shown to alter the expression of miRNAs and gene networks involved in metabolism, translation, cell cycle, and RBC cytoskeleton [9,16,17,18,19,20]. In addition to Bcl11a, Sox6, Klf1, and Tal1 are known to regulate HbF expression [21,22,23] but the mechanisms, direct or indirect, through which HU affects the expression at the mRNA or protein levels of these and other transcription factors are not fully elucidated.

At the cellular level, production of HbF is also poorly understood. Gamma-globin gene expression is silenced shortly after birth in all erythroid cells, except a small population of cells termed F-cells, which continue to exist throughout life. The HbF present in these cells represent less than 1% the amount of HbA produced in healthy individuals, but in the presence of chronic or acute erythroid stress, the number of F cells increases and total HbF production can rise considerably [15]. Treatment can increase production of HbF to up to 30% total hemoglobin or higher, but response to HU as well as distribution of HbF per F-cell varies among the treated patients [10]. Even in individuals with high lifetime HbF, as seen with hereditary persistence of fetal hemoglobin, the concentration of HbF per F-cell and distribution (pancellular versus heterocellular) can be highly variable [24].

As described in an accompanying report [25], we have quantified the frequencies by flow cytometry of nine populations of hematopoietic stem and progenitor cell (HSPCs), defined by their surface antigen profiles in 70 patients with SCD. This revealed that the hematopoietic system in SCD is deeply perturbed, as compared to that of healthy individuals. The main findings were that, regardless of treatment, SCD was associated with a 10- to 20-fold increase in the concentration of circulating CD34^dim^ cells, a two to five increase in CD34^bright^ cells (which contains mostly HSPCs), a depletion in committed erythroid progenitors, and an increase in the concentration of hematopoietic stem cells. We also observed novel populations of cells with a phenotype of LT-HSCs, with high levels of CD49f++ expression, as well as sub-populations of 235a expressing cells, which are nonexistent in healthy individuals. Treatment with HU was associated with a normalization in the concentration of all subpopulation of HSPCs, relative to that of controls, except for committed erythroid progenitors (MEPs), which were depleted. Importantly, we observed a global decrease in the concentration of all circulating HSPCs, per volume of blood, as a function of length of treatment with HU.

The concentration of each individual HSPC sub-populations in the peripheral blood is determined by its frequency in the bone marrow and its trafficking between the bone marrow, circulation, and other organs. Previous studies revealed that steady state SCD patients with low HbF levels had elevated levels of G-CSF [26]. More recently, Lamming et al. [27] observed that large increases in SCF, G-CSF, GM-CSF, and IL-8 were associated with dramatically higher concentration of HSCs during vaso-occlusive crises, which might be a form of spontaneous mobilization of these cells.

Here, we report that the proportions of circulating stem and progenitor cells are highly variable between individuals, but highly stable over time. We also report that these proportions of stem and progenitor cells are highly correlated with HbF levels in HU treated patients and with plasma concentrations of several chemokines.

## 2. Materials and Methods

### 2.1. IRB Approval

All samples were acquired under protocols approved by the Albert Einstein College of Medicine and Montefiore Medical Center internal review boards (IRB # 2017-8034, initial approval date: 11/20/2017.

### 2.2. Sample Acquisition

A total of 10 to 20-mL peripheral blood (PB) samples from 70 SCD patients (stratified into three treatment groups) and 20 healthy controls obtained by venipuncture in a yellow top tube (Sodium polyanethole sulfonate, acid citrate dextrose) are described in the accompanying article [25]. In addition, a second PB sample from a subset of these patients and controls (7 healthy African- American controls, 11 SCD patients treated with HU, and 12 treated by transfusion) were obtained 15 to 23 months after the initial collection. The clinical data from the patients whose blood was collected twice is described in Table S1.

As in the accompanying paper [25], mononuclear cells were isolated using Histopaque, as recommended by the manufacturer (Sigma-Aldrich, St Louis, MO, USA) and multiple aliquots were frozen in liquid nitrogen. Plasma samples were collected by centrifugation and stored in liquid nitrogen. Complete blood counts (CBCs) were performed using a Sysmex XN-9000 analyzer.

### 2.3. Flow Cytometry Analysis

Flow cytometry analysis was performed on a Cytek Aurora using the antibody panel described in Table S2.

Lineage markers consisted of a mixture of 10 antigens known to not be expressed in HSPCs and included CD2, CD3, CD4, CD7, CD8, CD10, CD14, CD19, CD20, CD56, and CD235a. All lineage markers except CD235a were labelled with biotin and detected with Streptavidin BV421. An FITC labelled anti-CD235a was used in order to assess the expression of this antigen, independent of the other lineage markers.

Data were analyzed using FloJo 10.6 (Ashland, OR, USA). Cells were first gated on forward and side scatter, to eliminate debris and doublets. Dead cells were gated out using a live/dead Zombie dye. Uniform gates were used throughout the experiments. At the time of analysis, gate positioning was established using concatenated FCS files. Once established, the gates were applied to each sample individually.

### 2.4. Cytokine Concentration Analysis

Plasma cytokine concentrations were analyzed using the multiplex kits from Millipore (Milliplex), using a panel of 65 chemokines and cytokines developed by Eve technology (Calgary, AB, Canada).

### 2.5. Statistical Analysis

All analysis were performed using R 3.6.3. Graphs and *p*-values were produced using the ggplot2 package. Regression analysis was performed using the glm R package, the lm R function, and other base R functions. Multiple regression analysis was performed using the broom, caret, and leaps R packages. The optimal number of parameters in the linear models was first evaluated with the leaps package, which also assesses the co-linearity of the variables. The broom and caret packages were then used to explore the models further.

## 3. Results

### 3.1. HbF Levels Are Correlated with the Percentages of HMCs and HPCs

**Figure 1:**
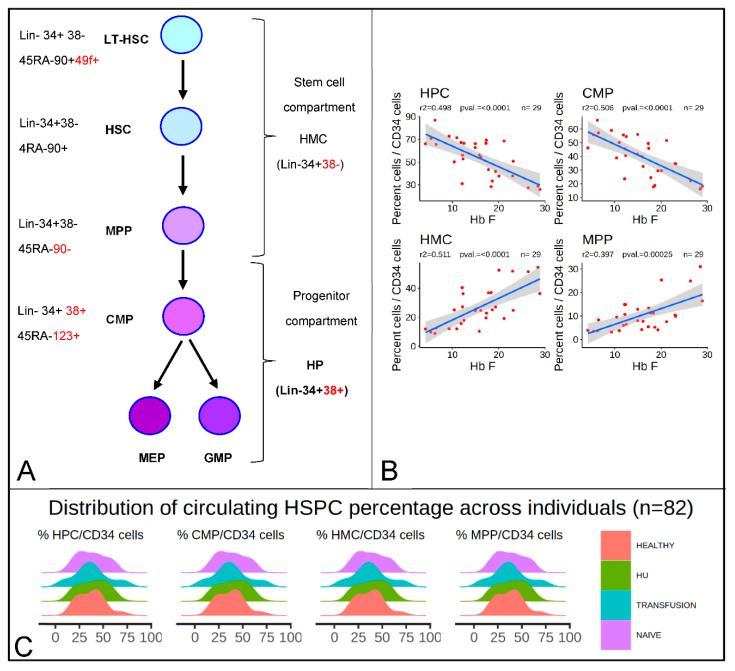
HbF levels are inversely proportional to the percentage of hematopoietic progenitors cells (HPCs), and common myeloid progenitors (CMPs) and proportional to the percentage of hematopoietic multipotent cells (HMCs) and multipotent progenitors (MPPs) in sickle cell disease (SCD) patients treated with hydroxyurea (HU). (**A**) Diagram illustrating the hematopoietic stem and progenitor hierarchy and the antigens used to define each subpopulation of hematopoietic stem and progenitor cell (HSPCs). (**B**) Regression analysis of the percentage of HbF as a function of the percentage of HPCs, CMPs, HMCs, and MPPs/CD34^bright^. Linear regression line is represented in blue. (*r*^2^ and F-statistic *p*-values are provided above). Grey smooth represents the 95% confidence interval. The percentage HbF is negatively correlated with the percentage of HPC/CD34 cells and positively correlated with the proportion of HMC/CD34. (**C**) Ridgeline density plots illustrating the broad distribution of the percentages of HMCs, HPCs, MPPs, and CMP/CD34^bright^ in controls and in SCD patients that are treatment-naïve, treated with HU, or treated with transfusion (*n* = 82).

As shown in Figure 1A, the human hematopoietic hierarchy is delineated into a stem and a progenitor cell compartment. Here, the cells in the stem cell compartment are referred to as hematopoietic multipotent cells (HMCs), which include 49f+ long-term hematopoietic stem cells (LT-HSCs), hematopoietic stem cells (HSCs), multipotent progenitors (MPPs), and lymphoid-primed multi-potential progenitors (LMPPs). Here, the progenitor compartments are referred to as hematopoietic progenitor cells (HPCs), which include common myeloid progenitors (CMPs), megakaryocyte-erythroid progenitors (MEPs), and granulocyte-monocyte progenitors (GMPs).

Analysis of the relationship between the concentration of individual subpopulations of HSPCs and the response to HbF in 26 HU patients, age 12 to 61 years, unexpectedly revealed a strong negative correlation (*r*^2^ = 0.47) between the HbF levels and the proportion of HPCs per CD34^bright^ cells (Figure 1B). Further examination of the data revealed that the correlation was even stronger and reached an *r*^2^ = 0.62 in outliers (*n* = 20), i.e., patients treated with HU for less than 30 months, were eliminated (Figure S1).

Since the proportion of HMCs and HPCs relative to the number of CD34^bright^ were roughly in inverse proportion, as these two cell populations are defined as the Lin^−^CD34^bright^ cells that differ by their expression of CD38, we also observed that the number of HMCs/CD34^bright^ was positively correlated with HbF (*r*^2^ = 0.47). Further analysis revealed that the inverse correlation of HPCs was driven mostly by CMPs (*r*^2^ = 0.50), whereas the positive correlation of HMCs was secondary to the increase of MPPs (*r*^2^ = 0.40), since other progenitor populations including HSCs, MEPs, and GMPs (Figure 1A) did not correlate significantly with HbF levels (data not shown). Therefore, individuals that exhibited high levels of CMPs and low levels of MPPs responded poorly to HU, whereas those who exhibited low levels of CMPs and high levels of MPPs responded strongly. This could indicate either that HU affected the proportions of HSPCs, or on the contrary, that the proportions of HSPCs affect the HbF response to HU.

### 3.2. The Proportion of HSPCs Is Stable over Long Periods of Time in Both Controls and SCD Patients

To differentiate between these hypotheses, we examined the distribution of the percentages of HSPC/CD34^bright^ cells in SCD patients grouped by treatment modality (naive, transfusion, or HU-treated) and in healthy controls (Figure 1C). This revealed that the distributions were very broad in all groups, with the percentages of HMCs, MPPs, HPCs, and CMPs ranging between 10% and 80%. High or low proportions of all sub-populations of HSPCs could be found in controls and in patients that were treated with HU or in the absence of HU, suggesting that the proportions of these cells might not be determined by treatment modality or even by the SCD genotype.

This analysis raised the question of the underlying cause of the variations in the proportion of HSPCs in circulation. As shown in the accompanying article [25], technical repeats clearly demonstrated that our flow cytometry measurements of the concentration of HSPCs were highly reproducible, suggesting that technical artifacts were not responsible for these variations. To determine if the proportion of HSPCs in the circulation was stable over time, or whether it varied widely in individuals in steady-state, we first examined a cohort of healthy volunteers from whom blood was collected twice in 2010, by the New York Blood Center, at 7- to 10-days intervals, to assess the effect of blood donation on hematopoiesis.

**Figure 2:**
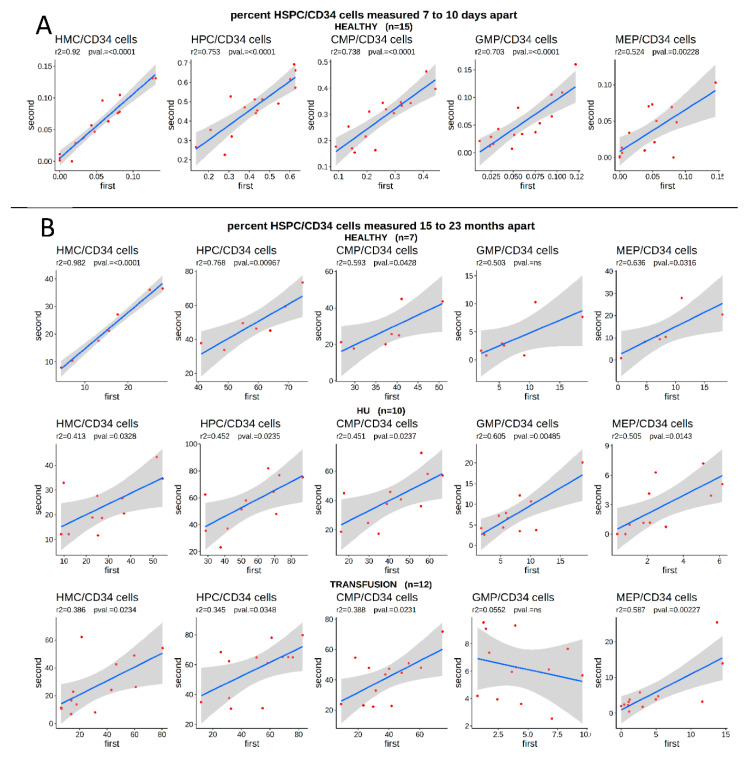
Percentage of circulating HSPC/CD34^bright^ cells in the blood is stable over time. (**A**) Blood samples were collected from 15 healthy individuals at 7 to 10 day intervals and the percentage of circulating HSPC/CD34^bright^ cells was measured by FACS. Blue line represents the linear regression; grey smooth area represents the 95% confidence intervals (*r*^2^ and F-statistic *p*-values are provided above). The percentage of circulating HSPCs was highly variable between individuals but stable over time within the same individual. X- and y-axis: Percentage of HSPC/CD34^bright^ cells measured at first and second collection. (**B**) Blood samples were collected from 7 healthy individuals, 10 HU-treated SCD patients, and 12 transfusion-treated SCD patients at 15- to 20-month intervals and the percentage of HSPCs was measured by FACS. The percentage of circulating HSPC/CD34^bright^ cells was highly variable between individuals, but stable over time in the same individuals, particularly in healthy individuals.

Within this cohort, the proportion of circulating HSPCs in the blood was also highly variable between individuals, but the values observed within the same individual were strongly correlated when the two samples collected at a 7- to 10-day interval were compared (Figure 2A). This clearly suggested that some healthy individuals showed persistently low levels of circulating HMCs and high levels of HPCs, while in others, the situation was reversed.

To expand upon this data, we collected a second sample from a subset of the SCD patients and healthy volunteers, 15 to 23 months after the initial blood draw, to determine if the number of HSPCs was stable over longer periods of time. Importantly, this revealed that while the number of unfractionated circulating CD34^bright^ cells/live cells varied over time (not shown), the proportion of HSPCs/CD34^bright^ cells in healthy individuals as well as in SCD patients, was surprisingly stable, as illustrated by the strong positive correlation between the proportion of all sub-populations, in healthy controls or in SCD patients, treated either with transfusion or with HU (Figure 2B).

This demonstrated that HU treatment or other aspects of SCD were not responsible for the variations in the percentage of HSPCs and therefore that the proportion of HMCs, HPCs, and their sub-populations might be a biomarker for HbF response to HU treatment.

**Figure 3:**
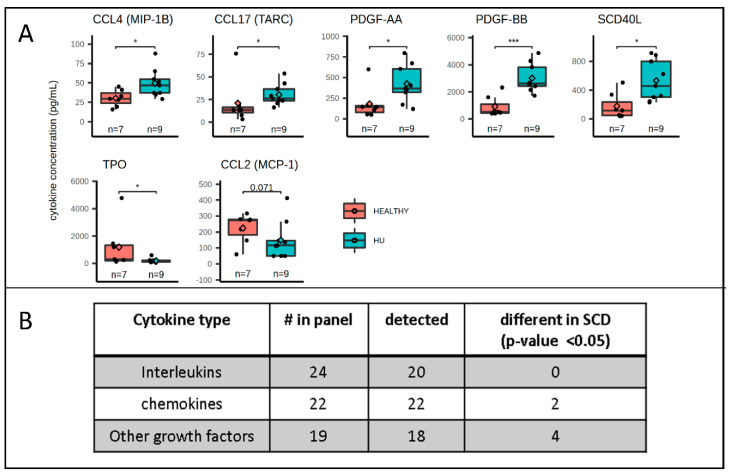
Concentrations of cytokine in the plasma of SCD and healthy individuals. (**A**) Boxplots summarizing the concentration of the 7 cytokines t present at significantly different concentrations in the plasma of the SCD patients compared to the controls. The box outlines the first and third quartiles. The horizontal bar represents the median; the diamond represents the mean. Significance was calculated using the Wilcox test (with FDR correction). * *p* ≤ 0.05; *** *p* ≤ 0.001. (**B**) Global results of the cytokine array. Plasma concentration of 65 cytokines in seven healthy controls and nine SCD patients on HU was assessed using FACS-based multiplex array methods.

The proportion of circulating HSPCs was correlated with the levels of 5 chemokines and PDGF-BB. The milieu of cytokines and chemokines controlled the overall level of hematopoietic activity, the differentiation rate of the various sub-population of HSPCs, and the trafficking of HSPCs between the bone marrow and circulation [28]. To determine if one or more cytokines or chemokines might control the proportion of circulating HSPCs, we measured the plasma concentrations of 65 cytokines/chemokines of eight healthy individuals and nine HU-treated SCD patients, using a commercial multiplex FACS-based assay. This revealed that the concentrations of several cytokines with chemokine activity (MIP-1B (CCL4), PDGF-AA, PDGF-BB, sCD40L, and TARC (CCL17)) were two- to three-fold higher in the plasma of the HU-treated patients, while the MCP-1 (CCL2) and TPO concentrations were lower (Figure 3A). The concentration of the remaining 60 out of 67 cytokines tested, including SCF, G-CSF, GM-CSF, IL-3, and IL-8, which were shown to be elevated in crisis [27], were undetectable or were detected at similar levels in the SCD patients and in healthy controls (Figure 3B and Table S3).

**Figure 4:**
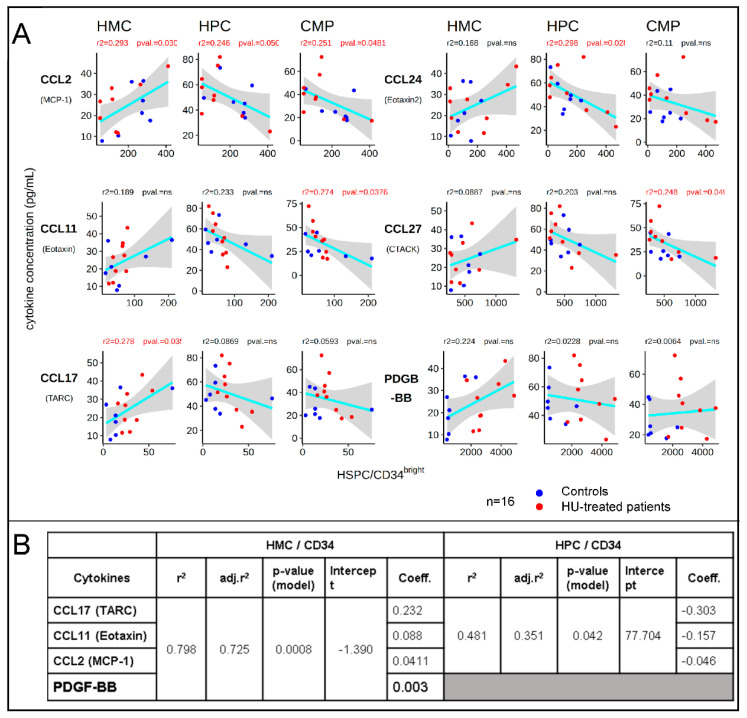
Correlation between plasma chemokine concentrations and HMC and HPC/CD34^bright^ cells. (**A**) Plots illustrating the linear correlations between the percentage of circulating HSPCs and the plasma concentration of the cytokines. Cyan line represents the linear regression; grey smooth area represents the 95% confidence intervals (*r*^2^ and *p*-values are provided above each graph). (**B**) Table illustrates the result of multiple regression analyses. A four-chemokine model explains about 80% (*r*^2^ = 0.798) of the variation in the percentage of HMC per CD34. A three-chemokine model explains about 50% (*r*^2^ = 0.481) of the variation in the percentage of HPC per CD34. In these models, CCL17 and CCL11 are the two most influential chemokines.

Analysis of the correlations between the plasma concentration of cytokines and the proportions of HPCs and HMCs in the peripheral blood revealed that 5 chemokines (CCL2, CCL11, CCL17, CCL24, and CCL27) had a significant correlation (*p* < 0.05) with the proportion of HMCs, HPCs, or CMPs per CD34^bright^ cells). PDGF-BB levels were also correlated with HMC/CD34^bright^, but at lower significance (*p* < 0.1). The coefficients of correlation (*r*^2^) were between 0.2 and 0.3, but more importantly, all correlations were positive with HMCs/CD34^bright^ and negative with HPCs/CD34^bright^ (Figure 4A). Of additional interest, two of these chemokines and PDGF-BB were among the seven cytokines that exhibited significantly different plasma concentrations in the HU-treated patients, as compared to the healthy controls (Figure 3B).

To determine the combination of cytokines that best explained the distribution of HMCs and HPCs, we then performed a multiple regression analysis between the proportion of HMCs or HPCs and all the cytokines that exhibited a correlation, with at least one of the subpopulation of HSPCs.

Remarkably, this revealed that, despite the relatively low *r*^2^ for each individual chemokine, a highly significant (*p* = 0.0008), four-chemokine model including CCL17 (TARC), CCL11 (Eotaxin), CCL2 (MCP-1), and PDGF-BB could explain about 80% of the variation in the proportion of HMC/CD34 in our cohort of SCD and healthy individuals (Figure 4B). A model based on three of these four chemokines could explain about 50% of the variation in HPC/CD34 or in CMP/CD34. Models including only CCL11 plus CCL2 (Eotaxin-2) had slightly higher *r*^2^ coefficients than the three cytokines model for the HPC/CD34. Of note, although CCL24 exhibited the highest correlation with proportion HPC/CD34, it was not part of any of the best multiple regression models.

**Figure 5:**
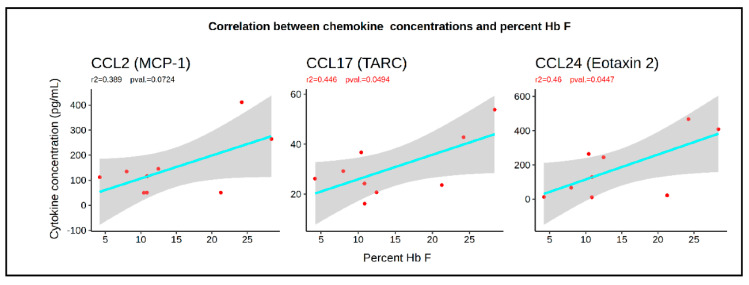
Correlation between HbF levels and the concentration of CCL2, CCL7, and CCL24. Plots illustrating the correlation, *r*^2^, and F-statistic *p*-values between HbF and the three chemokines.

Further analysis revealed that the plasma concentrations of CCL2, CCL17, and CCL24 were also significantly correlated with the HbF levels, with *r*^2^ in the 0.4 to 0.5 range (Figure 5). Together, these data suggest that these chemokines determine, in part, the concentration of HSPCs in the peripheral blood of healthy controls and SCD patients, and might have either a direct or indirect contribution to the HbF response.

## 4. Discussion

It was long known that the number of circulating CD34+ cells show significant variance between individuals, but these variations are multifactorial and are poorly understood. In our cohort, the proportion of each HSPC population, relative to the total number of circulating CD34^bright^ cells, varied more than 20-fold in both the SCD cohort and in healthy individuals. We observed that in some individuals, the circulating CD34^bright^ are comprised mostly of HPCs, while in others, HMCs predominate.

Unexpectedly, we observed strong correlations between the proportion of HMCs and HPCs with HbF levels in 26 patients treated with HU. These findings raised two questions: Why is the number of HSPCs so variable? Why is there a correlation between HSPC concentrations and HbF?

Our observation that the proportion of HSPCs were relatively stable over more than one year in both healthy individuals and SCD patients at a steady state, regardless of treatment type, suggests that neither the sole presence of SCD, nor the treatment modality, is the primary cause of inter-individual differences in peripheral HSPCs. Rather, these differences might be caused by epigenetic mechanisms or genetic polymorphisms that might be under selective pressure, as peripheral HSPCs are believed to have a functional role with recruitment to the sites of inflammation [29], and transit through organs such as the spleen, altering their specification [29,30].

We propose that genetically or epigenetically determined mechanisms set a baseline level of circulating HSPCs, either by controlling their rate of differentiation and self-renewal, or by controlling mobilization from the bone marrow.

The number of HSPCs in the circulation is known to be affected by pharmacological manipulations or by changes in the environment, such as inflammation or infection. However, whether the proportion of HMCs and HPCs is also altered, is unknown. Lamming et al. observed that the number of hematopoietic stem cells in the PB of SCD patients is higher during crisis than during steady state, likely in response to increased levels of mobilizing cytokines [27]. Several of our SCD patients had sickle cell crisis and infections, which likely temporarily increased the number of circulating HSPCs in between the two PB draws that were performed during this study. The stability of the proportion of HSPCs/CD34 in SCD patients that we nevertheless observed, therefore suggests that the mechanisms that control the proportion of HSPCs in the circulation were quite robust.

The determination of the plasma concentrations of 65 cytokines/chemokines yielded several important clues. The growth factors known to be involved in HSPC mobilization (G-CSF, GM-CSF, SCF, Flt-3, IL3, and IL11) [31] were either undetectable or were present at similar concentrations in the SCD patients and the controls. The low concentration of many of these factors in both SCD patients and healthy controls was not unexpected, as the plasma samples were collected at steady-state. By contrast, 5 members of the CC chemokines family (CCL2, CCL11, CCL17, CCL24, and CCL27) and PDGF-BB (a growth factor that also has important roles in chemotaxis [32]), correlated with the proportion of HMCs, HPCs, of CMPs, while none of the 30+ cytokines present in the panel exhibited any correlation. Remarkably, all correlations between the plasma concentration of these chemokines and the proportion of HMCs were positive, but negative with the proportion of HPCs, suggesting that these chemokines might control the differentiation of HMCs to HPCs, or the migration of these populations to the periphery.

Our observation that the levels of CCL2, CCL11, CCL17, and PDGF-BB, could explain 80% of the variability in the proportion of HMCs in SCD patients and the healthy controls, suggests that these chemokines contribute to setting the basal levels of HMCs and HPCs in the circulation. The levels of CCL17 and CCL24 (*p* < 0.05) and of CCL2 (*p* = 0.075) also correlated with the HbF levels. However, how these chemokines might control HbF response, is unclear.

The canonical function of chemokines is to activate or attract to the sites of inflammation effector cells, such as lymphocytes, monocytes, neutrophils, and eosinophils [33], but a subset of these proteins also regulate HSPCs [34,35,36,37]. CCL24, which specifically blocks the differentiation of myeloid progenitors [38] and CCL3, which binds the same receptors as several of the chemokines that we identified in this report, control myeloid lineage differentiation and the size of the HSPC pool [39]. CCR2, the receptor for CCL2 and CCL11, is expressed on HSPCs [40] and CCR2-positive HSCs were found to be the most upstream contributor to emergency myelopoiesis, after myocardial infarction [41]. Therefore, most of the chemokines that we found to be correlated with HSPCs were already implicated in the control of myeloid progenitor differentiation. Importantly, a genetic polymorphism in the CCL2 gene was associated with stroke and HbF variability in SCD [42].

Together, these data support the hypothesis that these chemokines might regulate the differentiation of stem cells into progenitor and erythroid precursor cells, and contribute to the determination of the level and the cellular distribution of HbF in SCD. Further studies are needed to elucidate the details of these mechanisms.

Some of the chemokines that we identified as correlating with HbF are disrupted in hematological malignancies, and agonist and antagonist molecules that interfere or promote interaction between these chemokines and their receptors were developed as anti-cancer drugs [43]. These molecules might prove useful to manipulate the HMC/HPC ratio and ultimately increase the HbF levels in SCD patients.

Measuring the concentration of the various HSPC populations is an internally-controlled, technically straightforward, FACS-based, single-tube flow cytometry method, which is highly amenable to clinical use and application. Our results suggest that the ratio of HMCs to HPCs, or of CMPs to MPPs, might be a useful biomarker to predict HbF response to HU in SCD patients. Markers that can predict HbF response might become increasingly clinically relevant, as alternative treatment modalities for SCD are becoming available.

## Supplementary Materials

The following are available online at https://www.mdpi.com/2073-4409/9/10/2199/s1. Figure S1: Regression analysis of the percentage of HbF as a function of the percentage of HPCs, CMPs, HMCs, and MPPs /CD34bright. Table S1: Clinical data. Table S2: Antibodies. Table S3: Plasma concentration of 65 cytokines in SCD and controls.
