# Supplementary material for: HbF Levels in Sickle Cell Disease Are Associated with Proportion of Circulating Hematopoietic Stem and Progenitor Cells and CC-Chemokines
Source: Cells. 2020 Sep 29;9(10):2199. doi: 10.3390/cells9102199 (PMC7650715; doi:10.3390/cells9102199)
Supplement: Supplementary file 1 [file cells-09-02199-s001.zip › Sup figures.pdf]

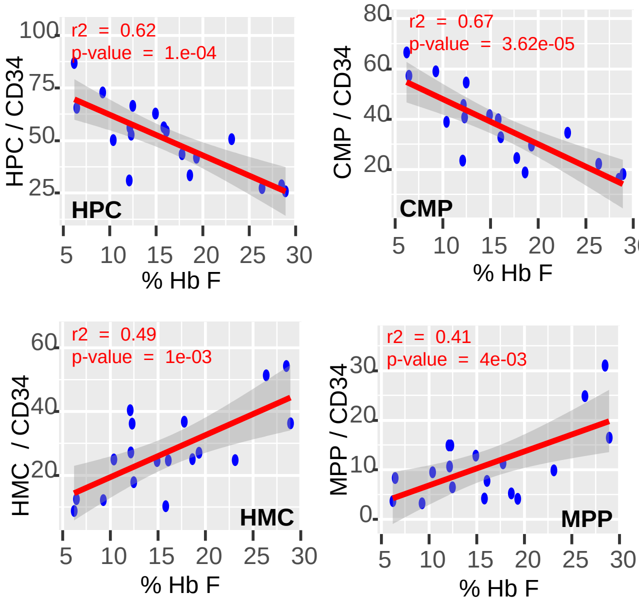

n= 20

Figure S1: *Regression analysis of the percentage of HbF as a function of the percentage of HPCs, CMPs, HMCs, and MPPs /CD34bright.* *Patients treated with HU for less than 30 months were excluded. Linear regression line is represented in red (r2 and F-statistic p-values are provided above). Grey smooth represents the 95% confidence for prediction for the linear models. The percentage HbF is negatively correlated with the proportion of HPC/CD34 and positively correlated with the proportion of HMC/CD34, n=20.*

Figure S1
