# Supplementary material for: HbF Levels in Sickle Cell Disease Are Associated with Proportion of Circulating Hematopoietic Stem and Progenitor Cells and CC-Chemokines
Source: Cells. 2020 Sep 29;9(10):2199. doi: 10.3390/cells9102199 (PMC7650715; doi:10.3390/cells9102199)
Supplement: Supplementary file 1 [file cells-09-02199-s001.zip › Table S1.docx]

| ID | Days between draw | Treatment | WBC  k/mL | | MCV  fL | | Platelet  k/uL | | Reticulocyte  % | | Hematocrit  % | | Hemoglobin  g/dL | | Hemoglobin F  % | |
| --- | --- | --- | --- | --- | --- | --- | --- | --- | --- | --- | --- | --- | --- | --- | --- | --- |
|  |  | **Draw** | **1st** | **2nd** | **1st** | **2nd** | **1st** | **2nd** | **1st** | **2nd** | **1st** | **2nd** | **1st** | **2nd** | **1st** | **2nd** |
| 1 | 660 | Transfusion | 7.5 | 6.7 | 84.7 | 85.6 | 234 | 192 | 13.2 | 8 | 30.9 | 35 | 10.8 | 11.5 | 0.5 | 0.5 |
| 2 | 555 | Transfusion | 10.2 | 6.5 | 79.1 | 83 | 240 | 171 | 5.0 | 3.2 | 32.1 | 32.8 | 10.9 | 10.8 | 1.1 | 1.3 |
| 3 | 382 | Transfusion | 16.8 | 13.6 | 85.8 | 87 | 244 | 166 | 14.6 | 14 | 30.3 | 30.8 | 10.3 | 10.1 | 1.7 | 0.7 |
| 4 | 272 | Transfusion | 7.5 | 6.0 | 85.9 | 88.2 | 126 | 164 | 8.1 | 9.1 | 29.2 | 28.5 | 10.1 | 9.4 | 2.5 | 1.0 |
| 5 | 239 | Transfusion | 8.5 | 5.9 | 86.6 | 87 | 506 | 396 | 6.8 | 2.5 | 26.4 | 30.8 | 8.8 | 10.2 | 1.7 | 1.6 |
| 6 | 411 | Transfusion | 11.3 | 17.1 | 93.5 | 84.8 | 435 | 373 | 11.7 | 15 | 28.8 | 32.8 | 9.7 | 10.9 | 2.8 | 2.8 |
| 7 | 440 | Transfusion | 10.2 | 15.3 | 95.5 | 87.3 | 511 | 439 | 13.7 | 7.7 | 29.7 | 27.6 | 9.5 | 9.2 | 3.3 | 1.9 |
| 8 | 245 | Transfusion | 18.1 | 16.8 | 89.9 | 88.7 | 356 | 341 | 11.3 | 12.7 | 27.2 | 28.3 | 9.5 | 9.4 | 7.7 | 9.9 |
| 9 | 456 | Transfusion | 12.4 | 13.9 | 94.2 | 88.2 | 440 | 512 | 10.1 | 17.3 | 30.9 | 25.4 | 10.3 | 8.6 | 6.2 | 6.4 |
| 10 | 454 | Transfusion | 9.5 | 14.2 | 89 | 89.1 | 159 | 412 | 23.9 | 13.4 | 25.5 | 26.9 | 8.6 | 8.7 | 1.6 | 1.0 |
| 11 | 428 | Transfusion | 10.7 | 12.1 | 90.5 | 84.6 | 426 | 480 | 9.8 | 8.6 | 27.7 | 26.4 | 9 | 8.7 | 1.7 | 2.1 |
| 12 | 468 | Transfusion | 8.1 | 20.4 | 86.9 | 87.5 | 154 | 387 | 14.2 | 9.9 | 23.9 | 23.7 | 7.7 | 8 | 1.3 | 1.1 |
| Average | | **Transfusion** | **10.9** | **12.4** | **88.5** | **86.8** | **319.3** | **336.1** | **11.9** | **10.1** | **28.6** | **29.1** | **9.6** | **9.6** | **2.3** | **2.5** |
| SD | | **Transfusion** | **3.3** | **4.8** | **4.4** | **1.8** | **136.0** | **123.2** | **4.6** | **4.4** | **2.4** | **3.2** | **0.9** | **1.0** | **2.4** | **2.7** |
| 1 | 419 | HU | 4.4 | 4.5 | 111.9 | 94.6 | 197 | 258 | 6 | 3.2 | 24.5 | 27.1 | 8.4 | 7.1 | 20.4 | 12.4 |
| 2 | 447 | HU | 5.9 | 7.1 | 102.3 | 106.1 | 608 | 265 | 3.3 | 4.4 | 25.4 | 24.4 | 8.7 | 8.4 | 22.3 | 23.2 |
| 3 | 356 | HU | 5.7 | 8.2 | 102.5 | 91.1 | 605 | 509 | 4.5 | 5.4 | 28.4 | 25.6 | 9.6 | 8.7 | 18.4 | 10.1 |
| 4 | 185 | HU | 9.3 | 10 | 103.3 | 103.9 | 372 | 341 | 18.2 | 15.3 | 24.8 | 26.9 | 8.9 | 9.6 | 16.2 | 21.1 |
| 5 | 356 | HU | 9.4 | 8.4 | 91.9 | 91.6 | 295 | 365 | 12 | 5.1 | 28.3 | 30.7 | 9.5 | 10.2 | 5.8 | 7.8 |
| 6 | 454 | HU | 8.6 | 11.2 | 105 | 108.7 | 373 | 498 | 4.2 | 15.2 | 20 | 21.2 | 7.1 | 7.4 | 28.2 | 9.2 |
| 7 | 214 | HU | 11.3 | 12 | 94.6 | 96.9 | 378 | 565 | 11.5 | 11.2 | 24.5 | 28.4 | 8.2 | 9.3 | 5.1 | 3.9 |
| 8 | 474 | HU | 10.7 | 8.5 | 90.3 | 91.3 | 241 | 550 | 16.3 | 22.9 | 20.4 | 23 | 7.2 | 8.1 | 12 |  |
| 9 | 452 | HU | 10.6 | 8.3 | 104.2 | 100.8 | 409 | 25.9 | 16.4 | 17.4 | 29.6 |  | 11 | 9.2 | 14.7 | 12.3 |
| 10 | 464 | HU | 9.1 | 13.3 | 96.4 | 98.3 | 324 | 233 | 10.8 | 15 | 26.5 | 23.1 | 9.6 | 8.4 | 18 | 19.7 |
| 11 | 313 | HU | 8.7 | 11.6 | 99.6 | 86.6 | 428 | 446 | 20.3 | 16.9 | 24.2 | 22.6 | 8.6 | 7.9 | 9.1 | 12.6 |
| Average | | HU | **8.5** | **9.4** | **100.2** | **97.3** | **384.5** | **368.7** | **11.2** | **12.0** | **25.1** | **25.3** | **8.8** | **8.6** | **15.5** | **13.2** |
| SD | | HU | **2.1** | **2.4** | **6.1** | **6.7** | **123.9** | **157.7** | **5.8** | **6.2** | **2.9** | **2.8** | **1.1** | **0.9** | **6.8** | **5.9** |

Table S1: Clinical data
