# Supplementary material for: HbF Levels in Sickle Cell Disease Are Associated with Proportion of Circulating Hematopoietic Stem and Progenitor Cells and CC-Chemokines
Source: Cells. 2020 Sep 29;9(10):2199. doi: 10.3390/cells9102199 (PMC7650715; doi:10.3390/cells9102199)
Supplement: Supplementary file 1 [file cells-09-02199-s001.zip › Table S2.docx]

| Antibodies | source | Cat # |
| --- | --- | --- |
| CD90-SB436(V2) | eBioscience | 62-0909-42 |
| CD45Ra-BV510 (V7) | Biolegend | 304142 |
| CD123-BV605 (V10) | Biolegend | 306026 |
| CD235a-FITC(B2) | eBioscience | 11-9987-82 |
| CD49f-PE (YG1) | eBioscience | 12-0495-83 |
| CD38-PE-Cy7 (YG9) | eBioscience | 25-0389-42 |
| CD34-APC (R1) | BD | 555824 |
| CD33-Alexa700 (R4 | eBioscience | 56-0338-42 |
| Zombie L/D (R7): | ThermoFisher |  |
| **Lineage antibodies** |  |  |
| CD2 (RPA-2.10), PE-Cyanine5 | ThermoFisher | 15-0029-42 |
| CD3 (UCHT1), PE-Cyanine5 | ThermoFisher | 15-0038-42 |
| CD4 (S3.5), PE-Cyanine5 | ThermoFisher | MHCD0406 |
| CD7 (CD7-6B7), PE-Cyanine5 | ThermoFisher | MHCD0706 |
| CD8 (3B5), PE-Cyanine5 | ThermoFisher | MHCD0806 |
| CD10 (CB-CALLA)), PE-Cyanine5 | ThermoFisher | 15-0106-42 |
| CD14 (TuK4), PE-Cyanine5 | ThermoFisher | MHCD1406 |
| CD19 (HIB19), PE-Cyanine5 | ThermoFisher | 15-0199-42 |
| CD20 (2H7) PE-Cyanine5 | ThermoFisher | 15-0209-42 |
| CD56 (MEM-188), PE-Cyanine5 | ThermoFisher | MHCD5606; |
| **Table S1: Antibodies** | | |
